# Brazilian Portuguese Adaptation of the Updated Clinical Frailty Scale: Cross‐Cultural Adaptation, Reliability, and Convergent Validity in Hospitalized Older Adults

**DOI:** 10.1155/jare/6605802

**Published:** 2026-05-30

**Authors:** Miguel K. Rodrigues, Ryan A. Jenkins, Vitor Malta, Carlos A. C. Lopes, Mayron F. Oliveira

**Affiliations:** ^1^ Physiotherapy Unit, VO_2_ Care, Physiotherapy Hospital Company & Care, Vila Nova Star Hospital, São Paulo, São Paulo, Brazil; ^2^ Exercise Physiology and Integrated Cardiopulmonary Research Group - EPIC Group, Exercise Science, Lyon College, Batesville, Arkansas, USA, lyon.edu

**Keywords:** aging, clinical frailty scale, convergent validity, cross-cultural adaptation, frailty, reliability, translation

## Abstract

**Background:**

Frailty is a predictor of adverse outcomes in older adults, yet contemporary Portuguese‐language tools for standardized frailty assessment remain limited. We translated, culturally adapted, and evaluated the updated clinical frailty scale (CFS, v2.0) for Brazilian Portuguese.

**Objectives:**

To assess inter‐ and intrarater reliability and convergent validity of the updated CFS in hospitalized older adults.

**Methods:**

This cross‐sectional psychometric study included patients aged 65 years or older admitted for elective surgery. Following a standardized cross‐cultural adaptation process, two independent examiners administered the updated Brazilian Portuguese CFS 60 min apart to assess inter‐rater reliability. Intrarater reproducibility was evaluated after 24 h. Participants also completed the SF‐36 quality‐of‐life questionnaire to examine convergent validity.

**Results:**

Ninety‐four hospitalized older adults were included. Inter‐rater reliability was excellent (ICC[2, 1] = 0.92, 95% CI 0.88–0.95). Weighted Cohen’s kappa indicated almost perfect ordinal agreement (κw = 0.96, 95% CI 0.83–1.00). Measurement error was low (SEM = 0.44; MDC95 = 1.22). Convergent validity was strong, demonstrating a significant monotonic association between CFS scores and SF‐36 domains (Spearman’s *ρ* = −0.786, *p* < 0.01). Exploratory descriptive analyses were conducted to illustrate the clinical context of frailty classification in this sample. These observations were not intended to assess predictive validity or support confirmatory inference.

**Conclusion:**

The updated Brazilian Portuguese version of the CFS demonstrates excellent reproducibility and strong convergent validity in hospitalized older adults. These results support its use as a reliable clinical and research tool for frailty assessment in hospitalized older adults, within the scope of the measurement properties evaluated.

## 1. Introduction

Frailty is a multidimensional clinical syndrome marked by diminished physiological reserves and heightened vulnerability to stressors in later life. In surgical and acute‐care contexts, greater frailty burden is associated with higher postoperative mortality, increased complications, and prolonged intensive care unit (ICU) stays, emphasizing the need for accurate and efficient risk stratification among older adults [1, 2]. The clinical frailty scale (CFS) is a brief, clinician‐rated instrument that integrates information on physical health, functional capacity, cognition, and social resources to provide a global judgment of fitness or frailty. Since its introduction, the CFS has been widely adopted across care settings and has demonstrated strong prognostic value for adverse outcomes in older populations [3].

In Brazil, a rapidly aging demographic and the growing exposure of older adults to elective and urgent procedures underscore the pressing need for standardized frailty assessment that can inform decision‐making, optimize perioperative planning, and support longitudinal research. Local evidence already suggests that even pre‐frailty elevates risks in cardiovascular surgical pathways, including readmissions and other adverse events [4, 5]. Yet, routine frailty screening in Portuguese‐speaking settings remains uneven, limiting clinical comparability and opportunities for multicenter collaboration.

Responding to advances in geriatric science and clinical practice, an updated version of the CFS has been released to better align level descriptors with contemporary use and to clarify guidance in common clinical scenarios. Building on our prior work translating and culturally adapting the original CFS into Brazilian Portuguese [6], we now aim to translate, culturally adapt, and evaluate selected measurement properties of the updated CFS (v2.0) for use in Brazil. Specifically, we assessed inter‐ and intrarater reliability and convergent validity against a widely used measure of health‐related quality of life and provide clinical context for the instrument’s use in the perioperative setting.

By providing a contemporary, reliable, and context‐appropriate tool, this study seeks to harmonize frailty assessment across Portuguese‐speaking services and to enable direct comparability with international research employing the updated CFS version.

## 2. Methods

The primary objective of this study was to translate, culturally adapt, and evaluate the reliability and convergent validity of the updated CFS v2.0 for Brazilian Portuguese. Analyses involving clinical outcomes were performed solely to descriptively characterize the sample and provide clinical context for frailty classification, and they were not intended as confirmatory, criterion‐validity, or predictive analyses.

This was a cross‐sectional psychometric, quantitative study enrolled older patients (aged 65 years or older) admitted to the hospital for elective surgery. Exclusion criteria included the inability to complete the study protocol and cognitive, speech, hearing, or comprehension limitations that could interfere with standardized assessment procedures. The study comprised two phases: (1) translation and cultural adaptation of the updated CFS into Brazilian Portuguese and (2) evaluation of selected measurement properties of the translated instrument.

Following translation and adaptation, the Brazilian Portuguese version of the updated CFS was reviewed by 30 health professionals for language clarity and grammatical adequacy. A comprehension questionnaire was used to rate item clarity on a 1‐to‐5 scale. The results of this step supported the final wording of the Brazilian Portuguese version, with no substantial conceptual modifications required after expert review.

**FIGURE 1:**
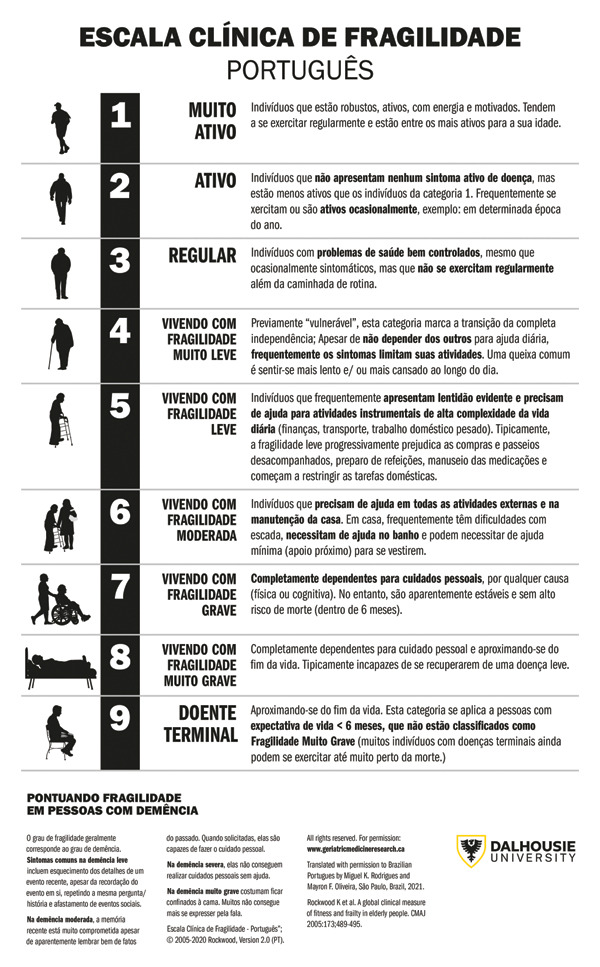
The clinical frailty scale.

After this evaluation, two questionnaires (CFS Figure 1 and SF‐36) were administered to patients upon hospital admission. To assess inter‐observer reproducibility, patients completed the updated CFS twice, administered by two different examiners (PT1 and PT2), with a 60‐min interval between administrations during the initial visit. Participants also completed the SF‐36, previously translated and validated in Brazil [7]. A second visit, scheduled 24 h later, evaluated intraobserver reproducibility in the same cohort. For descriptive purposes, participants were categorized according to established CFS thresholds. Individuals with CFS scores ≤ 3 were classified as nonfrail, whereas those with scores ≥ 4 were classified as frail, consistent with prior CFS validation studies and clinical use.

### 2.1. Ethical Statement

This study was approved by the Ethics Committee of Vila Nova Star Hospital, São Paulo, Brazil (approval number 4.915.914), in accordance with Resolution No. 466/2012 of the Brazilian National Health Council. All participants signed the informed consent form before inclusion in the study.

#### 2.1.1. Statistical Analysis

Statistical analysis was performed using SPSS version 20.0. Data distribution was assessed using the Kolmogorov–Smirnov test. Descriptive statistics are presented as mean ± standard deviation or median and interquartile range, as appropriate. Inter‐ and intrarater reliability of the CFS was assessed using the intraclass correlation coefficient (ICC). Inter‐rater reliability was evaluated using a two‐way random‐effects model with absolute agreement (ICC[2, 1]), as raters were considered representative of a broader clinical population. Intrarater reliability was assessed using the appropriate single‐measure ICC model. Ninety‐five percent confidence intervals were calculated for all ICC estimates. In addition, weighted Cohen’s kappa was calculated to assess ordinal agreement between ratings. Convergent validity was assessed using Spearman’s rank correlation coefficient (*ρ*) to examine the monotonic association between CFS scores and SF‐36 domains, given the ordinal or quasi‐ordinal nature of both instruments.

Exploratory subgroup analyses comparing frail and nonfrail participants were conducted for descriptive purposes only. Due to small subgroup sizes and non‐normal distributions, nonparametric tests were applied. The Mann–Whitney *U* test was used for ICU length of stay, and Fisher’s exact test was used for ICU admission rates. Measurement error was quantified using the standard error of measurement (SEM) and the minimal detectable change (MDC), calculated based on the reliability estimates.

Effect sizes were reported using rank‐biserial correlation for continuous outcomes and odds ratios with 95% confidence intervals for categorical outcomes. Statistical significance was set at *p* < 0.05.

## 3. Results

A total of 94 hospitalized older adults were included in the analysis. Most participants reported regular medication use and presented multiple comorbidities. Baseline characteristics are presented descriptively in Table 1 and should be interpreted as sample characterization rather than inferential comparisons between frailty groups. Analysis of the SF‐36 questionnaire demonstrated lower scores in physical health domains compared with mental and social domains (Table 1).

**TABLE 1:** Baseline characteristics of all study participants.

	All patients (*n* = 94)	Nonfrail (*n* = 49)	Frail (*n* = 45)
Anthropometrics			
Female, *n* (%)	44 (46.8%)	24 (49.0%)	20 (44.4%)^∗^
Age, years	75 ± 6	74 ± 5	79 ± 4^∗^
Weight, kg	73 ± 19	72 ± 5	76 ± 3
Height, m	1.66 ± 0.10	1.68 ± 0.11	1.66 ± 0.05
BMI, kg/m^2^	25.9 ± 5.0	25.0 ± 1.0	28.0 ± 2.0
ASA, points	2.04 ± 0.35	2.00 ± 0.30	2.20 ± 0.40
Comorbidities			
Smokers, *n* (%)	12 (12.8%)	3 (6.1%)	9 (20%)^∗^
Previous smokers, *n* (%)	14 (14.9%)	5 (10.2%)	9 (20%)^∗^
Hypertension, *n* (%)	52 (55.3%)	34 (69.4%)	18 (40%)^∗^
Diabetes, *n* (%)	23 (24.5%)	5 (10.2%)	18 (40%)^∗^
Dyslipidemia, *n* (%)	41 (43.6%)	14 (28.6%)	27 (60%)^∗^
Medications/previous disorders			
Use medication, *n* (%)	89 (94.7%)	44 (89.8%)	45 (100%)
Antihypertensive, *n* (%)	52 (55.3%)	34 (69.4%)	18 (40%)^∗^
Beta blockers, *n* (%)	28 (29.8%)	19 (38.8%)	9 (20%)
Diuretics, *n* (%)	17 (18.1%)	8 (16.3%)	9 (20%)
Cardiovascular disorder, *n* (%)	46 (48.9%)	19 (38.8%)	27 (60%)^∗^
Previous abdominal surgery, *n* (%)	60 (63.8%)	24 (49.0%)	36 (80%)^∗^
Basic hemodynamics and blood samples			
Hemoglobin, g/dL	11.4 ± 3.0	11.3 ± 0.9	11.6 ± 0.4
Hematocrit, *n* (%)	34.50 ± 9.0	34.2 ± 3.0	35.2 ± 1.0
Urea, mmoL/L	39.1 ± 6.3	40.5 ± 4.5	33.2 ± 8.3
Creatine, mg/dL	1.15 ± 0.60	1.25 ± 0.10	0.75 ± 0.25
pH	7.37 ± 0.55	7.38 ± 0.62	7.35 ± 0.77
PaO_2_, mmHg	129 ± 75	131 ± 32	123 ± 10
PaCO_2_, mmHg	39 ± 7	40 ± 5	36 ± 9
HCO_3_, mmoL/L	24.0 ± 2.50	23.0 ± 1.0	25.5 ± 0.5
SpO_2_, %	93.0 ± 13.0	92.0 ± 6.0	98.0 ± 1.0^∗^
SF‐36 QoL Questionnaire			
Physical, points	45 ± 8	50 ± 3	46 ± 6^∗^
Emotional, points	53 ± 7	59 ± 6	50 ± 9
Social, points	47 ± 5	46 ± 6	47 ± 5
Mental health, points	48 ± 8	52 ± 4	46 ± 8^∗^
Vitality, points	40 ± 7	42 ± 4	39 ± 8
General health, points	52 ± 9	53 ± 8	44 ± 7^∗^

*Note: n*: number of patients; %: percentage; kg: kilogram; m: meter; g/dL: grams per deciliter; mmoL/L: millimoles per liter; L: liter; mg/dL: milligrams per deciliter; pH: hydrogen ion concentration; PaO_2_: partial pressure of arterial oxygen; PaCO_2_: partial pressure of arterial carbon dioxide; HCO3: bicarbonate; SpO_2_: arterial oxygen saturation measured by pulse oximetry; mmHg: millimeters of mercury. Values are expressed as mean ± standard deviation or *n* (%).

Abbreviations: ASA, American Society of Anesthesiologists; BMI, body mass index; QoL, quality of life.

^∗^Indicates a significant (*p* < 0.05) comparison between nonfrail and frail groups.

Comparison of CFS assessments showed high consistency between raters and across repeated assessments. Mean CFS scores were similar between examiner 1 (PT1) and examiner 2 (PT2), as well as across repeated evaluations by the same examiner. Inter‐rater reliability was excellent, as demonstrated by a two‐way random‐effects ICC with absolute agreement (ICC[2, 1] = 0.92, 95% CI 0.88–0.95). The SEM was 0.44, and the MDC at the 95% confidence level (MDC95) was 1.22. Intrarater reliability was similarly high. Weighted Cohen’s kappa indicated almost perfect ordinal agreement between ratings (kw = 0.96, 95% CI 0.83–1.00).

For descriptive and illustrative purposes, participants were stratified into nonfrail (*n* = 49) and frail (*n* = 45) groups according to established CFS classification thresholds. This stratification was performed to characterize the clinical profile of the cohort in a hospitalized setting and was not intended for confirmatory or predictive inference. Descriptive subgroup comparisons involving ICU‐related variables were explored only as secondary contextual observations and should be interpreted with caution, particularly given the exploratory design and subgroup imbalance.

Convergent validity analysis demonstrated a strong and statistically significant monotonic association between CFS scores and SF‐36 physical and functional health domains, indicating that higher frailty levels were associated with lower health‐related quality of life (Spearman’s *ρ* = −0.786, *p* < 0.01).

## 4. Discussion

The present study demonstrates that the Brazilian Portuguese version of the updated CFS v2.0 exhibits excellent inter‐ and intrarater reproducibility, as well as supportive evidence of convergent validity, in hospitalized older adults undergoing elective procedures. These findings support its use as a standardized frailty assessment instrument within the scope of the measurement properties examined in this study. The SEM and MDC values further suggest that small variations in CFS scores may reflect measurement error, whereas larger differences are more likely to represent true clinical changes.

The existing literature consistently demonstrates that frailty can predict health outcomes, particularly adverse events in older adults [8]. Frail individuals are characterized by functional and physiological decline, exhibiting poorer prognoses when subjected to stressful events such as elective surgery [9]. They are more susceptible to falls, hospitalization, readmissions, institutionalization, and mortality compared to their age‐matched counterparts [10, 11]. These findings underscore the critical importance of reliable frailty assessment tools in healthcare settings. Accurate frailty evaluation enables healthcare providers to identify high‐risk patients and support clinical decision‐making.

Recent years have seen an increase in surgical procedures among older adults. While some patients experience favorable clinical courses, others develop severe postoperative complications, including increased mechanical ventilation use, leading to prolonged ICU stays and diminished quality of life [12]. Our group has recently demonstrated that frail and pre‐frail patients experience higher rates of mechanical ventilation, adverse events, and extended hospital stays [4, 5]. Furthermore, older individuals with higher frailty degrees exhibit significantly higher mortality rates, increased postoperative complications, prolonged ICU stays, and decreased quality of life following surgical procedures [13, 14].

Although ICU‐related subgroup observations were explored in this study, these analyses were secondary, descriptive, and not part of the primary psychometric objectives. Accordingly, they should not be interpreted as evidence of predictive validity and are reported only to provide clinical context for the hospitalized sample.

### 4.1. Clinical Implications

By demonstrating reproducibility and supportive evidence of convergent validity, this study provides clinicians in Brazil with a standardized instrument for frailty assessment in hospitalized older adults undergoing elective procedures. Incorporating the CFS into routine evaluations may support clinical judgement, perioperative planning, and communication across teams when frailty screening is considered relevant [15]. Because the present sample was restricted to hospitalized older adults admitted for elective surgery, extrapolation to other populations, including community‐dwelling individuals, emergency admissions, and more severely frail patients, should be made with caution.

The translation and validation of instruments like the CFS into Brazilian Portuguese could greatly benefit healthcare practices in Portuguese‐speaking regions, facilitating more accurate and comprehensive assessments of frailty among older populations. This highlights the importance of ongoing research efforts aimed at adapting and validating frailty assessment tools across diverse cultural and linguistic contexts.

### 4.2. Study Limitations

This study has some limitations. Although the sample size was adequate for reliability assessment and convergent validity and it is consistent with previous studies evaluating similar instruments, no formal a priori sample size or power calculation was performed. Therefore, the precision of the reliability estimates should be interpreted with caution.

The study population consisted of hospitalized older adults undergoing elective procedures, which may represent a relatively selected and clinically specific subgroup of patients. As such, the findings may not be generalizable to broader geriatric populations, including community‐dwelling older adults, emergency surgical patients, or individuals with more advanced frailty.

Some baseline differences observed between frailty groups in Table 1 may appear counterintuitive and should be interpreted cautiously, as these comparisons were descriptive rather than etiologic or predictive and may reflect sample‐specific characteristics and subgroup heterogeneity. The exclusion of patients with cognitive impairment represents an additional limitation. Although this decision was intended to support more standardized assessment conditions, it may have introduced selection bias and may limit applicability to real‐world geriatric populations, in whom cognitive impairment often coexists with frailty.

Convergent validity was assessed using the SF‐36, which measures health‐related quality of life rather than frailty directly. Therefore, the observed associations should be interpreted as indirect evidence of convergent validity, and the absence of a frailty‐specific comparator represents a limitation of the study. The 24‐h interval used for repeated assessment may have increased risk of recall bias and, consequently, may have overestimated intrarater reliability. However, in hospitalized older adults, longer intervals could also increase the likelihood of true clinical change between assessments.

Finally, exploratory analyses of ICU‐related variables were not designed or powered to assess predictive validity and should be interpreted only as descriptive contextual observations.

## 5. Conclusion

The Brazilian Portuguese version of the updated CFS demonstrates excellent reproducibility and supportive evidence of convergent validity in hospitalized older adults undergoing elective procedures. It may serve as a useful standardized instrument for frailty assessment in Brazilian Portuguese‐speaking clinical and research settings within the scope of the measurement properties examined here. Further studies including broader and more diverse populations are warranted to expand evidence on its applicability.

## Author Contributions

All authors contributed to all stages of the study. The contributions include study design and conceptualization, data collection, data analysis, manuscript writing, and critical manuscript revision. M.K.R. and M.F.O. were involved in the elaboration of the study; M.K.R., V.M., and C.A.C.L. were involved in data collection; M.K.R., R.A.J., V.M., C.A.C.L., and M.F.O. were involved in data analysis; M.K.R., R.A.J., V.M., and C.A.C.L. were involved in manuscript drafting; and M.F.O. was involved in critical revision of the final version.

## Funding

No financial support was received in this manuscript.

## Disclosure

All authors declare that they participated sufficiently in the work to take public responsibility for its content. Each author substantially contributed to the study design, data collection and analysis, drafting, and critical revision of the manuscript. All authors have approved the final version of the manuscript and agree with its submission to the Journal of Aging Research.

## Conflicts of Interest

The authors declare no conflicts of interest.

## Data Availability

The data that support the findings of this study are available on request from the corresponding author. The data are not publicly available due to privacy or ethical restrictions.
